# Hesperidin ameliorates bleomycin-induced experimental pulmonary fibrosis via inhibition of TGF-beta1/Smad3/AMPK and IkappaBalpha/NF-kappaB pathways

**DOI:** 10.17179/excli2019-1094

**Published:** 2019-08-29

**Authors:** Zheng Zhou, Amit D. Kandhare, Anwesha A. Kandhare, Subhash L. Bodhankar

**Affiliations:** 1 Department of Respiratory Medicine, the Second Affiliated Hospital of Zhengzhou University, Zhengzhou City, Henan Province, 450014, China; 2 Department of Pharmacology, Center for Advanced Research in Pharmaceutical Sciences, Bharati Vidyapeeth Deemed University, Poona College of Pharmacy, Pune-411 038, India

**Keywords:** AMPK, bleomycin, hesperidin, IkappaBalpha, NF-kappaB, Nrf2, pulmonary fibrosis, Smad3, TGF-beta1

## Abstract

Bleomycin (BLM) is a chemotherapeutic agent which is associated with Idiopathic pulmonary fibrosis (IPF) due to its chronic administration. Hesperidin, a bioflavonoid has been reported to possess antioxidant, anti-inflammatory, wound healing, and antiapoptotic potential. To evaluate the therapeutic potential of hesperidin against BLM-induced pulmonary fibrosis and decipher its possible mechanism of action. Intraperitoneal administration of BLM (6 IU/kg) caused induction of IPF in Sprague-Dawley rats. Rats were treated with hesperidin (25, 50, and 100 mg/kg, p.o.) for 28 days, followed by estimation of various parameters in bronchoalveolar lavage fluid (BALF) and lung. Hesperidin (50 and 100 mg/kg) administration significantly ameliorated (*p < *0.05) alterations induced by BLM in lung index, percent oxygen saturation, serum ALP and LDH levels, BALF differential cell count, and lung function test. Elevated levels of oxido-nitrosative stress, hydroxyproline, and myeloperoxidase levels in BALF and lung were significantly decreased by hesperidin on day 14. Hesperidin significantly inhibited BLM-induced down-regulated lung Nrf2 and HO-1 as well as up-regulated TNF-α, IL-1β, IL-6, collagen-1, TGF-β, and Smad-3 mRNA expressions. Western blot analysis showed that alteration in lung NF-κB, IκBα, AMPK, and PP2C-α protein expressions were ameliorated by hesperidin on day 28. Furthermore, BLM induced histological and ultrastructural aberrations in the lung which were attenuated by hesperidin treatment. Hesperidin alleviates BLM-induced IPF via inhibition of TGF-β1/Smad3/AMPK and IκBα/NF-κB pathways which in turn ameliorate the modulation of oxido-inflammatory markers (Nrf2 and HO-1) and pro-inflammatory markers (TNF-α, IL-1β, and IL-6) to reduce collagen deposition during pulmonary fibrosis. See also Figure 1(Fig. 1).

## Abbreviations

Adenosine monophosphate-activated protein kinase (AMPK), Alkaline Phosphatase (ALP), Bleomycin (BLM), Bronchoalveolar Lavage Fluid (BALF), Enhanced Pause (Penh), Expired Volume (EV), Frequency of breathing (f), Glyceraldehyde 3-phosphate dehydrogenase (GAPDH), Glutathione (GSH), Heme oxygenase 1 (HO-1), Hydroxyproline (HP), Idiopathic pulmonary fibrosis (IPF), Interleukins (IL's), Lactate Dehydrogenase (LDH), Malondialdehyde (MDA), Mothers against decapentaplegic homolog-3 (Smad-3), Myeloperoxidase (MPO), Nitric Oxide (NO), Non-phosphorylated AMPK (PP2C-α), Nuclear factor E2-related factor 2 (Nrf2), Nuclear factor-Kappa B (NF-κB), Nuclear factor of kappa light polypeptide gene enhancer in B-cells inhibitor-alpha (IκBα), Transforming Growth Factor-β (TGF-β), Transmission Electron Microscopy (TEM), Tumor Necrosis Factor-alpha (TNF-α).

## Introduction

Idiopathic Pulmonary fibrosis (IPF) is a multifactorial, chronic disease featured by progressive formation of a scar in the lung parenchyma. The prognosis and diagnosis of IPF are poor with 3-5 years of median survival rate after its diagnosis, which leads to high mortality and morbidity (De Langhe et al., 2015[8]). It is expected that almost 5 million individuals suffer from IPF, and estimated prevalence of IPF is 13-20 per 100,000 people worldwide (Raghu, 2017[45]). Lung fibrosis is mainly characterized by injury to the epithelial cell (ECs), deposition of excessive collagen and extracellular matrix (elastic fibers, proteoglycans, fibronectin and fibrillar collagens) in the alveolar walls, myofibroblast accumulation and matrix remodeling resulting in distorted alveolar structure, lung parenchyma remodeling, and progressive alteration in lung function (Della Latta et al., 2015[9]). An array of factors contributed to induction and maintenance of acute lung injuries including cigarette smoke, industrial dust, aspiration, sepsis, sarcoidosis, pneumonia and recurrent exposure to various drugs and chemical agents (including antibiotics, chemotherapeutic agents, immunosuppressant) which led to pulmonary edema, inflammation, and lung fibrosis (Khaefi et al., 2017[29]; Neisi et al., 2018[43]).

Accumulated evidence suggested that elevated influx of reactive oxygen species (ROS) due to pulmonary insults resulted in the release of pro-inflammatory cytokines (such as tumor necrosis factor-α (TNF-α) and interleukins (IL's)) during initial stage (Kandhare et al., 2015[23]). These cause apoptosis of alveolar epithelial cell and basement membrane denudation leading to increased transition of epithelial cells to mesenchyme. Alteration in pulmonary architecture, i.e., abnormal pulmonary remodeling resulted in impaired gaseous exchange across lung alveoli (Bargagli et al., 2009[2]). Furthermore, transforming growth factor-beta (TGF-β), which is a profibrotic factor, has been implicated in the differentiation of myofibroblast from fibroblast (Fernandez and Eickelberg, 2012[13]). Excess formation of myofibroblast results in elevated synthesis and deposition of extracellular matrix (ECM) proteins, including type I collagen and fibronectin into lungs (Fernandez and Eickelberg, 2012[13]). TGF-β has been reported to stimulate ROS generation via Smad 2/3 and mitogen-activated protein kinase (MAPK) signaling activation (Saito et al., 2018[49]). TGF-β also plays an indisputable role in the inhibition of adenosine monophosphate-activated protein kinase (AMPK) activity to induce epithelial cell injury (Saito et al., 2018[49]). Thus, novel therapeutic strategies should focus on targeted modulation of these biomarkers for effective management for IPF.

The current treatment regimens for the management of IPF include various agents such as cytokine antagonists, immunosuppressant (methotrexate), anti-inflammatory agents (corticosteroid), and colchicines which mainly focus on suppressing the inflammatory response (Raghu, 2017[45]). Additionally, the two recognized medicines for IPF, namely pirfenidone and nintedanib, which are potent inhibitors of TGF-β and tyrosine-kinase respectively, inhibit collagen production (Yoon et al., 2018[64]). However, these treatment regimens are associated with long-term side effects such as elevated alanine aminotransferase and aspartate aminotransferase activity, abnormalities in skin and gastrointestinal functions which limit their efficacy for clinical implication (Raghu et al., 2015[46]). Moreover, these treatments provide partial relief from disease progression in only a fraction of patients or delay the failure of pulmonary function (Kim et al., 2015[31]). Despite a tremendous increase in the growth of the pharmaceutical industry, the development of safe and effective agents for management of IPF remains an imperative challenge. In recent years, moieties from natural origin are gaining more importance as an alternative medicine for various disease states. Recently, isolated moieties from herbal origin such as Withaferin A, Trigoneoside Ib, Quercetin, Resveratrol, Curcumin, and Berberine have been documented to ameliorate pulmonary fibrosis (Kilic et al., 2014[30]; Verma et al., 2013[56]).

Bleomycin, a chemotherapeutic agent of glycopeptide group of antibiotics, is used clinically for the management of an array of human malignancies such as cervical cancer, testicular cancer, Hodgkin's disease, and squamous cell carcinomas of the head and neck (Liu et al., 2016[39]). However, chronic administration of bleomycin (BLM) resulted in induction of pulmonary fibrosis. Bleomycin hydrolase, bleomycin inactivating enzyme, exhibits low levels in the pulmonary area making lungs more prone to bleomycin-induced IPF (Jóna et al., 2016[19]). A growing body of evidence suggests that BLM has the ability to interact with oxygen to form pseudo-enzyme, which generates free radicals (such as superoxide and hydroxide) (Kandhare et al., 2015[23]). The elevated levels of free radicals cause overproduction of reactive oxygen species (ROS) which induce break-down of double-strand DNA and thus interrupt the cell cycle resulting in pulmonary toxicity (Chaudhary et al., 2006[4]). In rodents also, intratracheal instillation of BLM resulted in elevated ROS, oxidative stress, nitric oxide, inflammatory influx (neutrophils and macrophages) and cytokines (TNF-α, ILs) production via activation of Nuclear factor-Kappa B (NF-κB). Activation of these vicious mediators cause epithelial cell injury, fibroblast proliferation, parenchymal inflammation, basement membrane damage and collagen deposition thereby promoting fibrosis in interstitial and intra-alveolar regions of lung (Kilic et al., 2014[30]; Verma et al., 2013[56]).

Hesperidin (5,7,3'-trihydroxy-4'-methoxy flavanones, hesperitin-7-O-rutinoside) is a bioflavonoid glycoside widely present in citrus fruits. Hesperidin has been documented to possess a wide range of pharmacological properties including antioxidant, antidiabetic, anti-inflammatory, wound healing, neuroprotective, antihypertensive, antiarthritic, cardioprotective, hepatoprotective, anticancer and antiapoptotic potential (Ding et al., 2018[11]; Eghtesadi et al., 2016[12]; Haddadi et al., 2017[16]; Maneesai et al., 2018[41]; Salden et al., 2016[50]; Shahbazi et al., 2018[53]; Wu et al., 2015[59]; Yu et al., 2016[65]). Researchers showed that hesperidin significantly attenuated oxidative DNA damage *in vivo* (Yamamoto et al., 2008[60]). It has been reported that administration of hesperidin in ovalbumin-challenged experimental animals significantly attenuated airway hyperresponsiveness via enhanced pause (Penh) and inhibition of inflammatory influx (Yang et al., 2012[62]). Furthermore, evidence suggested that hesperidin inhibit transforming growth factor-β TGF-β/Smad3 signaling pathway and thus reduce collagen-1 expression (Wu et al., 2015[59]). Previous researchers documented the potential of hesperidin against various pulmonary maladies (Ding et al., 2018[11]). However, its efficacy against BLM-induced pulmonary fibrosis has not yet been evaluated. Hence, the present investigation is aimed to evaluate the potential and possible mechanism of action of hesperidin against BLM-induced pulmonary fibrosis.

## Materials and Method

### Animals

Sprague-Dawley rats (adult male, 180-220 g) were procured from the National Institute of Biosciences, Pune (India). The housing conditions for rats throughout the experimental protocol were: temparature: 24 ± 1 °C, relative humidity: 45-55 %, dark/light cycle: 12:12 h, food: standard pellet chow, water: filtered (*ad libitum*). A time of 09:00 to 17:00 h were consider to carry out all the experiments protocol which was approved by the Institutional Animal Ethics Committee (IAEC, Poona College of Pharmacy). A guidelines mentioned by Committee for Control and Supervision of Experimentation on Animals (CPCSEA), Government of India were followed to perform all experiments

### Chemicals and Kits

Hesperidin (Sigma Chemical Co., St. Louis, MO/USA), BLM (Biochem Pharmaceutical Industries Limited, India), Total RNA Extraction kit (MP Biomedicals India Private Limited, India) and One-step RT-PCR kit (MP Biomedicals India Private Limited, India), TNF-α, IL's (IL-1β, and IL-6) ELISA kits (enzyme-linked immunosorbent assay, Rat specific, Bethyl Laboratories Inc., Montgomery, TX/USA), primary antibodies of Thr-172 (phosphorylated AMPK), PP2C-α (non-phosphorylated AMPK), p-NF-κB and p-IκBα (Abcam, Cambridge, MA/USA).

### Induction of lung fibrosis and drug treatment

BLM hydrochloride (6 IU/kg in 0.9 % NaCl) was used to induce PF in overnight fasted rats according to method report elsewhere (Kandhare et al., 2015[23]), then they were divided randomly into the various groups (n=16) viz., Normal group (treated with distilled water (DW, 10 mg/kg)), Sham control group (received intratracheal saline and treated with DW (10 mg/kg)), BLM control group (received intratracheal BLM and treated with DW (10 mg/kg)), MP treated group (received intratracheal BLM and treated with methylprednisolone (10 mg/kg)), Hesperidin treated group (received intratracheal BLM and treated with either hesperidin (25 or 50 or 100 mg/kg), per se treated group (treated with hesperidin (100 mg/kg)). 

A previous report used to determine the treatment doses of hesperidin (25, 50, 100 mg/kg) (Li et al., 2018[36]; Visnagri et al., 2014[57]) and methylprednisolone (10 mg/kg) (Cortijo et al., 2009[7]; Li and Cui, 2002[37]). Rats were treated with either vehicle or hesperidin or methylprednisolone for 28 days after BLM administration.

### Lung function and peripheral blood oxygen measurements

The respiratory dynamics were evaluated by using Whole-body flow-through plethysmography (EMKA Technologies, France) whereas *in vivo* peripheral blood oxygen content were determined by a peripheral pulse Ox sensor (ChoiceMMed, V1.0CF3, MD300CF3, China) according to method described elsewhere (Kandhare et al., 2015[23]). 

### Serum biochemistry 

On day 28, blood was withdrawn by a retro-orbital puncture and the levels of serum Alanine transaminase (ALT), Aspartate Aminotransferase (AST), Alkaline phosphatase (ALP) and lactate dehydrogenase (LDH) were measured by using reagent assay kits (Accurex Biomedical Pvt. Ltd., Mumbai, India).

### BALF (Bronchoalveolar Lavage Fluid) and lung biochemical analysis

BALF total cell counts and levels of total protein, SOD, GSH, MDA, NO, hydroxyproline (HP), myeloperoxidase (MPO) in BALF and lung were estimated according to earlier reported methods (Kandhare et al., 2013[24], 2012[26]). Part of tissue samples (n=4) were stored at -70 °C for Reverse Transcription Polymerase Chain Reaction (RT-PCR) analysis and Western blot assay of various markers. Lung tissues from each group (n=3) were processed for histopathological examination, and one tissue from each group was processed for Transmission Electron Microscopy (TEM) examination.

### Reverse transcriptase (RT)-PCR and Western blot assay

The mRNA expressions of TNF-α, IL-1β, IL-6, Nrf2, HO-1, TGF-β, Collagen-1, Smad-3, and β-actin were analyzed in lung tissue using RT-PCR according to method described elsewhere (Kandhare et al., 2014[28]). Whereas, protein expressions of Thr-172 (phosphorylated AMPK), PP2C-α (non-phosphorylated AMPK), p-NF-κB and p-IκBα (nuclear factor of kappa light polypeptide gene enhancer in B-cells inhibitor-alpha), and GAPDH (Glyceraldehyde 3-phosphate dehydrogenase) were estimated in lung tissue according to method described elsewhere (Liang et al., 2019[38]).

### Histological and electron microscopic analysis

Histopathological analysis of lung tissue was carried out using hematoxylin and eosin (H&E) stain (on day 14) and Masson's trichrome (MT) and Picro-Sirius red (PSR) stains (on day 28) as described previously (Kandhare et al., 2015[23]). Whereas, on day 28 the lung ultrastructural studies were performed under a transmission electron microscope (H-7000 Hitachi) according to method described previously (Kandhare et al., 2015[23]).

### Statistical analysis

GraphPad Prism 5.0 software (GraphPad, San Diego, CA/USA) was used to perform data analysis. Data are expressed as mean ± standard error mean (SEM) and analyzed by using One-Way ANOVA followed by Tukey's multiple range post hoc analysis (for parametric tests) as well as Kruskal-Wallis test for post hoc analysis (non-parametric tests). A value of *p < *0.05 was considered to be statistically significant.

## Results

### Body weight and lung index

In BLM controlled rats, the body weight decreased significantly (*p < *0.05) and lung index increased significantly (*p < *0.05) when compared with normal and sham rats. Administration of MP (10 mg/kg) significantly (*p < *0.05) attenuated BLM-induced decrease in body weight and increase in lung index as compared to BLM controlled rats. When compared with BLM controlled rats, hesperidin (25, 50 and 100 mg/kg) treatment significantly (*p < *0.05) inhibited alterations in lung index and body weight. However, when compared with hesperidin treatment, MP (10 mg/kg) treatment more significantly (*p < *0.05) inhibited BLM-induced alterations in body weight and lung index (Table 1(Tab. 1)).

### Percent oxygen saturation

There was no significant difference in the percent oxygen saturation in normal, sham, and per se treated rats. However, when compared with normal as well as sham rats, there was significant decrease (*p < *0.05) in percent oxygen saturation in BLM controlled rats after intratracheal instillation of BLM. MP (10 mg/kg) administration significantly (*p < *0.05) increased percent oxygen saturation level as compared to BLM controlled rats. Administration of hesperidin (25, 50 and 100 mg/kg) also significantly increased (*p < *0.05) the level of percent oxygen saturation as compared to BLM controlled rats. However, inhibition of BLM-induced decrease in percent oxygen saturation level was more significant (*p < *0.05) in MP (10 mg/kg) as compared to hesperidin treatment (Table 1(Tab. 1)).

### Serum ALP and serum LDH levels

The serum ALP and LDH did not differ significantly in normal, sham, and per se treated rats. However in BLM controlled rats, the level of serum ALP and LDH elevated significantly (*p < *0.05) when compared with normal and sham rats. MP (10 mg/kg) administration significantly attenuated (*p < *0.05) BLM-induced increase in serum ALP and LDH as compared to BLM controlled rats. Hesperidin (50 and 100 mg/kg) significantly reduced (*p < *0.05) BLM-induced increase in serum ALP and LDH when compared with BLM controlled rats. BLM-induced increase in serum ALP and LDH was more significantly (*p < *0.05) decreased by MP (10 mg/kg) as compared to hesperidin treatment (Table 1(Tab. 1)). 

### Lung function test

When compared with normal and sham rats, administration of BLM caused significant alterations (*p < *0.05) in lung function test in BLM controlled rats. However, MP (10 mg/kg) treatment significantly inhibited (*p < *0.05) lung function test alterations induced by BLM when compared with BLM controlled rats. Hesperidin (50 and 100 mg/kg) also significantly attenuated (*p < *0.05) BLM-induced alterations in lung function test as compared to BLM controlled rats. BLM-induced alterations in PIF and enhanced paused were more significantly (*p < *0.05) decreased by treatment with hesperidin (100 mg/kg) when compared with MP (10 mg/kg) treatment. There was no any significant difference in the lung function test between sham, per se and normal rats (Table 2(Tab. 2)).

### BALF differential cell count

There was a significant increase (*p < *0.05) in the BALF total cell count, lymphocyte and eosinophil counts whereas neutrophil and monocyte count decreased significantly (*p < *0.05) in BLM controlled rats when compared with normal and sham rats. MP (10 mg/kg) treatment significantly inhibited (*p < *0.05) BLM-induced BALF differential cell count alterations as compared to BLM controlled rats. There was significant increase (*p < *0.05) in BALF neutrophil and monocyte counts, and significant decrease (*p < *0.05) in BALF total cell count, lymphocyte and eosinophil counts in hesperidin (50 and 100 mg/kg) treated rats as compared to BLM controlled rats. The attenuation of BLM-induced increased lymphocytes and eosinophils, whereas decreased monocytes counts in BALF was more significant (*p < *0.05) in MP (10 mg/kg) treated rats when compared with hesperidin treated rats (Table 3(Tab. 3)).

### SOD, GSH, MDA, NO and HP levels in BALF

Intratracheal instillation of BLM resulted in a significant (*p < *0.05) decrease in SOD and GSH levels in BALF as well as significant increase (*p < *0.05) in MDA, NO and HP levels in BALF of BLM controlled rats when compared with normal and sham rats. Whereas, MP (10 mg/kg) administration significantly (*p < *0.05) decreased the levels of MDA, NO, and HP in BALF and significantly increased (*p < *0.05) BALF SOD and GSH levels as compared to BLM controlled rats. The BLM-induced decrease in SOD and GSH levels increased significantly (*p < *0.05) in hesperidin (50 and 100 mg/kg) treatment whereas the levels of BALF MDA, NO and HP decreased significantly (*p < *0.05) in hesperidin (25, 50 and 100 mg/kg) treatment as compared to BLM controlled rats. Hesperidin (100 mg/kg) treatment more significantly (*p < *0.05) elevated BALF SOD and GSH levels as compared to MP (10 mg/kg) treatment. However, there was no significant difference in the BALF SOD, GSH, MDA, NO, and HP in normal, sham, and per se treated rats (Table 4(Tab. 4)).

### SOD, GSH, MDA, NO, HP and MPO in the lung

When compared with sham and normal rats, the levels of SOD and GSH significantly decreased (*p < *0.05) whereas levels of MDA, NO, HP and MPO increased significantly (*p < *0.05) in the lungs of BLM controlled rats after intratracheal instillation of BLM. MP (10 mg/kg) treatment showed significant (*p < *0.05) inhibition in BLM-induced alterations in SOD, GSH, MDA, NO, and HP in the lung as compared to BLM controlled rats. However, it failed to produce any significant reduction in elevated MPO levels compared to BLM controlled rats. Treatment with hesperidin (50 and 100 mg/kg) also significantly increased (*p < *0.05) SOD and GSH levels whereas significantly decreased (*p < *0.05) levels of MDA, NO, HP and MPO in the lungs as compared to BLM controlled rats. BLM-induced alterations in SOD, GSH, MDA, NO, and HP was more significantly (*p < *0.05) attenuated by MP (10 mg/kg) as compared to hesperidin treatment (Table 5(Tab. 5)).

### Nrf2, HO-1, TNF-α, IL-1β and IL-6 mRNA expression in the lung

Lung Nrf2 and HO-1 mRNA expressions was significantly down-regulated (*p < *0.05) whereas TNF-α, IL-1β, and IL-6 mRNA expressions significantly up-regulated (*p < *0.05) in BLM-controlled rats when compared with sham and normal rats. When compared with BLM controlled rats, treatment with MP (10 mg/kg) significantly (*p < *0.05) down-regulated lung TNF-α, IL-1β, and IL-6 mRNA expressions whereas it significantly (*p < *0.05) up-regulated lung Nrf2 and HO-1 mRNA expressions. BLM-induced alterations in lung Nrf2, HO-1, TNF-α, IL-1β, and IL-6 mRNA expressions were significantly attenuated (*p < *0.05) when rats treated with hesperidin (50 and 100 mg/kg) as compared to BLM controlled rats. The attenuation of alteration in mRNA expressions of lung TNF-α, IL-1β, IL-6, Nrf2 and HO-1 were more significant (*p < *0.05) in rats treated with MP (10 mg/kg) when compared with treatment with hesperidin (Figure 2(Fig. 2)). 

### Collagen-1, TGF-β and Smad-3 mRNA expressions in the lung

There was no significant difference in the lung collagen-1, TGF-β, and Smad-3 mRNA expressions in normal, sham, and per se treated rats. However, intratracheal instillation of BLM resulted in significant up-regulation (*p < *0.05) in lung collagen-1, TGF-β, and Smad-3 mRNA expressions in BLM controlled rats compared to sham and normal rats. Whereas, treatment with MP (10 mg/kg) significantly (*p < *0.05) inhibited BLM-induced up-regulated lung collagen-1, TGF-β, and Smad-3 mRNA expressions as compared to BLM controlled rats. Hesperidin (50 and 100 mg/kg) treatment also significantly down-regulated (*p < *0.05) mRNA expressions of collagen-1, TGF-β and Smad-3 in lungs when compared with BLM controlled rats. Administration of MP (10 mg/kg) showed more significant inhibition (*p < *0.05) in BLM-induced up-regulated lung collagen-1, TGF-β, and Smad-3 mRNA expressions as compared to hesperidin treatment (Figure 3(Fig. 3)).

### NF-κB and IκBα protein expressions in lung

There was significant up-regulation (*p < *0.05) in lung NF-κB and IκBα protein expressions in BLM controlled rats after intratracheal instillation of BLM when compared with sham and normal rats. Administration of MP (10 mg/kg) significantly inhibited (*p < *0.05) BLM-induced up-regulated lung NF-κB and IκBα protein expressions as compared to BLM controlled rats. Hesperidin (50 and 100 mg/kg) treatment also significantly down-regulated (*p < *0.05) lung NF-κB and IκBα protein expressions as compared to BLM controlled rats. However, BLM-induced up-regulated lung NF-κB, and IκBα protein expressions were more significantly (*p < *0.05) down-regulated by MP (10 mg/kg) as compared to hesperidin treatment. There was no significant difference in the lung NF-κB and IκBα protein expressions in normal, sham, and per se treated rats (Figure 4A and B(Fig. 4)). 

### AMPK and PP2C-α protein expressions in lung

When compared with sham and normal rats, lung AMPK was significantly down-regulated (*p < *0.05) whereas lung PP2C-α protein expression was significantly up-regulated (*p < *0.05) after BLM administration in BLM controlled rats. Treatment with hesperidin (50 and 100 mg/kg) significantly attenuated (*p < *0.05) BLM-induced down-regulated (*p < *0.05) lung AMPK and up-regulated (*p < *0.05) lung PP2C-α protein expressions when compared with BLM controlled rats. However, administration of MP (10 mg/kg) did not produce any significant inhibition in BLM-induced altered lung AMPK and PP2C-α protein expressions as compared to BLM controlled rats. The lung AMPK and PP2C-α protein expressions did not differ significantly in the normal, sham, and per se treated rats (Figure 4C and D(Fig. 4)). 

### Lung histology

The normal, sham and per se treated rats showed the well-organized histological architecture of lung tissue without any inflammatory infiltration and interstitial fibrosis (Figure 5.1A, 5.1B, 5.1G, 5.2A, 5.2B, 5.2G, 5.3A, 5.3B, and 5.3G(Fig. 5)). However, there was a significant increase (*p < *0.05) in inflammatory influx in the peribronchial and perivascular region, interstitial fibrosis, and Ashcroft score in BLM controlled rats as compared to normal and sham rats (Figure 5.1C, 5.2C, and 5.3C(Fig. 5)). MP (10 mg/kg) significantly inhibited (*p < *0.05) BLM-induced inflammatory infiltration, interstitial fibrosis, and Ashcroft score as compared to BLM controlled rats (Figure 5.1D, 5.2D, and 5.3D(Fig. 5)). Hesperidin (50 and 100 mg/kg) treatment also significantly decreased (*p < *0.05) peribronchial and perivascular inflammatory influx, interstitial fibrosis, and Ashcroft score as compared to BLM controlled rats (Figure 5.1E, 5.1F, 5.2E, 5.2F, 5.3E, and 5.3F(Fig. 5)). However, lung tissue from hesperidin (25 mg/kg) treated rats showed the presence of inflammatory infiltration and interstitial fibrosis (Figure 5H, I and J(Fig. 5)).

### Lung ultrastructural morphology

Ultrastructural studies of lung sections from normal rats showed the presence of a normal architecture of type II pneumocyte, nuclei, cytoplasm and bronchial epithelial cells. It showed normal nucleus of epithelial alveoli with the presence of some vacuolization and phagocytes (Figure 6A(Fig. 6)). Lung tissue from sham and per se treated rats showed the normal architecture of pneumocyte and intestinal septa with presences of few erythrocytes (Figure 6B and 6F(Fig. 6)). BLM-controlled rat showed presence of vacuolated type II alveolar cells, abnormal cytoplasm with shrunken nucleus and extensive deposition of collagen (Figure 6C(Fig. 6)). It was also evident with ballooning of endothelial, epithelial cells in the capillary lumen with lamellar bodies in its lungs. The interstitial septa were thickened and showed the presence of pyknosis of the alveolar epithelial cell, vesicular cytoplasm, and electron dense mitochondria (Figure 6C(Fig. 6)). Lung section from MP (10 mg/kg) administered rats showed the presence of normal nuclear membrane with mild collagen and cytoplasmic vesicular granules in vesicles (Figure 6D(Fig. 6)). Lung section of hesperidin (100 mg/kg) treated rat showed the presence of few inflammatory cells, an enlarged vesicle with cytoplasmic vesicular granules, and the normal architecture of type II pneumocyte and bronchial epithelial cells (Figure 6E(Fig. 6)).

## Discussion

Idiopathic pulmonary fibrosis (IPF) is a chronic, progressive interstitial lung disease resulting from the accumulation of excessive collagen, epithelial cell injury, matrix remodeling, and disruption of alveolar architecture leading to declining pulmonary function. Bleomycin (BLM), which is an effective antineoplastic agent, selectively induces pulmonary fibrosis. Thus, BLM is a well-established and widely used animal model to induce lung fibrosis, which resembles with the clinical characteristics of human IPF. In the present investigation also intratracheal instillation of BLM produced inflammatory infiltration in Bronchoalveolar lavage fluid (BALF) and lung, alteration in gas exchange and pulmonary function, elevated levels of ROS, and activation of TGF-B/Smad3 pathway which resulted in pulmonary fibrosis. However, administration of hesperidin significantly attenuated BLM-induced pulmonary fibrosis via inhibition of elevated oxido-nitrosative stress, the release of pro-inflammatory cytokines (TNF-α, ILs) and IκBα/NF-κB as well as modulation of TGF-B/smad3 pathway to reduce collagen deposition.

It has been well documented that animal models play a vital role in the evaluation of disease progression during BLM-induced PF (Kandhare et al., 2015[23], 2011[27]). Intratracheal instillation of BLM in the experimental murine model is associated with clinical characteristics of IPF, including a reduction in body weight and appetite, difficulty in breathing. Elevated inflammatory influx after BLM administration resulted in the deposition of collagen into the lung epithelial cells (Della Latta et al., 2015[9]). It caused an alteration in the integrity of the alveolar-capillary membrane, which in turn affected the efficiency of gas exchange across this membrane (Kandhare et al., 2015[23]; Liu et al., 2016[39]). Thus, determination of the oxygenated level of arterial hemoglobin is considered as hallmark of arterial lung disease (Collins et al., 2015[6]). Pulse Ox, i.e., the percentage of hemoglobin saturated with oxygen is an important but indirect marker for determination of peripheral blood oxygen content (Kandhare et al., 2015[23]). Previous reports suggested that BLM instillation led to decrease in the body weight and pulse Ox with an increase in the lung weight of rats (Kandhare et al., 2015[23]). In the present investigation, we have also documented similar findings after BLM administration, whereas treatment with hesperidin significantly attenuated these BLM-induced alterations in body weight, lung weight, and Pulse Ox.

Numerous evidence suggested that insult to pulmonary tissue induced by various toxicants resulted in an alteration in the respiratory patterns (Kandhare et al., 2019[25]; Mukherjee et al., 2017[42]). Therefore, lung function test (LFT) is considered as an important diagnostic tool for evaluation of respiratory patterns and severity of the pulmonary injury. An array of researchers have employed whole-body plethysmograph as a non-invasive method for analysis of respiratory patterns which provides an advantage of real-time analysis compared to biochemical and pathologic biomarkers of lung injury (Kandhare et al., 2019[25]; Mukherjee et al., 2017[42]). Amongst the various LFTs, tidal volume and breathing frequency reflected the acute response to toxins, thus earning the title of physiologic measures of ventilation. Whereas, inspiratory time, expiratory time, and enhanced pause evaluate the breath structure and are known as flow-derived parameters (Jacono et al., 2006[17]). Enhanced pause is used to determine the alterations in shape of airflow pattern which reflected the changes in elasticity and resistivity of lungs in response to inhaled toxins (Jóna et al., 2016[19]; Kandhare et al., 2015[23]). Furthermore, IPF is also associated with pulmonary vascular remodeling, which results in the development of pulmonary hypertension (Lettieri et al., 2006[34]). Clinically it has also been shown that IPF patients exhibit the characteristics of pulmonary hypertension, which significantly worsen the survival (Lettieri et al., 2006[34]). Previous investigators showed that installation of toxicants such as BLM, OVA or silica resulted in alterations in PFT, pulmonary arterial hypertension and right ventricular hypertrophy in experimental animals (Bei et al., 2013[3]; Schroll et al., 2010[51]). In the present investigation, administration of hesperidin significantly attenuated BLM-induced alteration in flow-derived parameters, pulmonary arterial hypertension, and right ventricular hypertrophy. The outcomes of this study is in accordance with results of earlier investigators which suggest that hesperidin attenuated ventricular hypertrophy via modulation of oxidative stress, TNF-α and TGF-β1 levels (Yu et al., 2016[65]).

Heme oxygenase-1 (HO-1) is a stress-inducible protein that plays a crucial role in the protection against oxidative stress and regulation of lung inflammation (Mahmoud et al., 2017[40]). Researchers documented that heme, which is a potent oxidant, converted to biliverdin in the presence of HO-1. Thus, it serves as an important catalyst in this rate-limiting step of oxidative insult (Kandhare et al., 2015[23]; Lakari et al., 2001[33]). Degradation of heme by HO-1 resulted in the formation of bile pigments, which possess potent antioxidant, anti-inflammatory, and antiapoptotic properties, which in turn ameliorated lung fibrosis (Fredenburgh et al., 2007[14]). Furthermore, mice, as well as patients deficient in HO-1 enzymatic activity, showed susceptibility to inflammatory diseases (Dennery, 2014[10]). Thus, researchers suggested that HO-1 plays an important role in the inhibition of fibrogenesis and amelioration of pulmonary fibrosis (Otterbein et al., 1999[44]). HO-1 is expressed in alveolar macrophages as well as type II pneumocytes, and its expression is induced by pro-inflammatory cytokines. Clinical and experimental evidence showed that there was a down-regulated expression of HO-1 in alveolar macrophages during interstitial lung disease (Kandhare et al., 2015[23]; Lakari et al., 2001[33]). In the present investigation, BLM-control animals exhibited a down-regulated expression of HO-1, whereas treatment with hesperidin significantly ameliorated this down-regulation of HO-1 expression. The findings of this study corroborates with outcomes of previous researchers indicated hesperidin improved cellular antioxidant levels through the modulation of HO-1 expression (Chen et al., 2010[5]).

Earlier studies have documented the significant role of pro-inflammatory cytokines (such as TNF-α and IL's) in the pathogenesis of BLM-induced pulmonary fibrosis (Bale et al., 2018[1]; Kandhare et al., 2015[23]). Cytokines have an ability to induce the production of fibroblast, synthesis of extracellular matrix, hyperplasia of goblet cell, production, and hypersecretion of mucus (Bale et al., 2018[1]). Macrophage has been suggested as an important source of these damaging pro-inflammatory cytokines (Lakari et al., 2001[33]; Yang et al., 2012[62]). Evidences demonstrated that BLM stimulates alveolar macrophages to release pro-inflammatory cytokines *in vitro *(Joshi et al., 2017[20]). Furthermore, NF-κB, which is a transcription factor from Rel protein family, regulates the transcription of an array of cytokine genes, including TNF-α and IL's. The IκBα (nuclear factor of kappa light polypeptide gene enhancer in B-cells inhibitor-alpha) is an enzyme complex that inhibits NF-κB and keeps them in an inactive state in the cytoplasm (Li et al., 2017[35]). Pulmonary damage due to the instillation of BLM results in phosphorylation of IκBα at Ser32/36 end, which further translocates NF-κB into the nucleus (Li et al., 2017[35]). This increased activity of NF-κB, in turn, mediated the influx of pro-inflammatory cytokines and thus activated the levels of pro-fibrotic markers (such as TGF-β1) (Chaudhary et al., 2006[4]). Researchers reported that exposure of BLM activates the ROS and RNS which further stimulate the expression of redox-sensitive transcription factor NF-κB and thus trigger inflammatory response via regulation of pro-inflammatory signaling (Li et al., 2017[35]). It has also been demonstrated that TNF-α induces pulmonary inflammation via the induction of apoptosis (Kuroki et al., 2003[32]). The results of the present investigation are in accordance with previous findings where instillation of BLM caused ROS influx which in turn phosphorylated IκBα and activated NF-κB resulting in the release of pro-inflammatory cytokines (TNF-α, IL-1β, and IL-6) and brought out pulmonary fibrosis. However, hesperidin significantly attenuated BLM-induced recruitment of ROS into lungs and BALF, thus decreasing the expression of TNF-α, IL-β, and IL-6 via inhibition of phosphorylation of IκBα and release of NF-κB. The results of the present study are in agreement with previous reports, which show that hesperidin ameliorates pulmonary injury via inhibition of NF-κB activation and elevated pro-inflammatory cytokine (TNF-α, IL-1β, and IL-6) response (Shahbazi et al., 2018[53]; Yeh et al., 2007[63]).

It has been well accepted that BLM-induced IPF associates with biphasic events where a “switch” appears between inflammatory and fibrotic phase at around day 14 after intratracheal instillation of bleomycin (Bale et al., 2018[1]; Kandhare et al., 2015[23]). The early inflammatory phase comprises of inflammatory influx, release of ROS and pro-inflammatory cytokines which are documented at day 14 whereas fibro-proliferative phase becomes significant from day 21 which is reflected by increase in collagen deposition in the alveolar spaces in response to proliferation and activation of fibroblasts (Cortijo et al., 2009[7]; Tashiro et al., 2017[55]). Transforming growth factor β1 (TGF-β1) which is a pro-fibrogenic cytokine, plays a vital role in an array of cellular functions including adhesion, migration, proliferation, and apoptosis (Willis and Borok, 2007[58]). Elevated levels of ROS caused up-regulation in TGF-β1 expression, which has an ability to induce proliferation of myofibroblasts from fibroblasts, which further contributes to the pathogenesis of pulmonary fibrosis (Yang et al., 2017[61]). Additionally, TGF-β1 also modulates several signaling pathways, including activation of Smad, TNF-α and IL-1β, inhibition of AMPK activity, which induces EC injury and extracellular matrix production, thus promoting the progression of pulmonary fibrosis. Researchers showed that transcription of collagen mRNA significantly increased after the accumulation of extracellular matrix via stimulation of TGF-β1 (Zhao et al., 2002[66]). Consistent with previous investigations, results of the present study also showed that BLM administration significantly elevated TGF-β mRNA expression in BLM control rats as compared to normal and sham rats. This TGF-β mRNA expression was significantly down-regulated in the hesperidin treated rats which may have further stimulated cascades of events including inhibition of Smad, TNF-α, and IL-1β which could be attributed to the pulmonary protective potential of hesperidin. A previous investigator also showed that hesperidin treatment significantly inhibited TGF-β induced non-small cell lung cancer and the result of the present study is in accordance with findings of the investigator (Yu et al., 2016[65]).

Smad3, a member of mothers against decapentaplegic homolog 3 has been implicated as a central player in the formation of the transcriptional activation complex that induces lung fibrosis (Zhao et al., 2002[66]; Zhao and Geverd, 2002[67]). Lung injury induced by toxicant such as BLM stimulates of TGF-β response, which further activates the type I receptor. This activation results in phosphorylation of Smad-2 and Smad-3 along with the formation of a heteromeric complex with Smad-4 (Gao et al., 2013[15]; Ji et al., 2013[18]). The translocation of this complex into the nucleus further promotes synthesis of type I collagen. During the development of pulmonary fibrosis, phosphoinositide-3-kinase-protein kinase B (PI3K-Akt) signaling pathway caused activation of phosphorylation of Smad2 and deactivation of Smad3 (Zhao and Geverd, 2002[67]). Numerous evidence suggested that hydroxyproline is an indirect measure of type I collagen synthesis and its excess deposition in the form of extracellular matrix reflected as a hallmark of pulmonary fibrosis (Kandhare et al., 2015[23]; Zhao and Geverd, 2002[67]). In the present study, intratracheal instillation of BLM resulted in down-regulated Smad3 mRNA expression and interestingly, this expression was significantly up-regulated by hesperidin which might have, in turn, created a negative feedback loop that down-regulated TGF-β signaling and collagen-I mRNA expression to ameliorate BLM-induced IPF. Previous investigators also reported the protective efficacy of hesperidin via modulation of TGF-β1/Smad3 signaling pathways (Mahmoud et al., 2017[40]) thus, the results of the present study provide a credential to the findings of these investigators (Mahmoud et al., 2017[40]).

Recently a study has proved that adenosine monophosphate-activated protein kinase (AMPK) is one of the key elements in the pathogenesis of fibrosis in various organs, including pulmonary fibrosis (Rangarajan et al., 2018[47]). Activation of AMPK is associated with a decrease in phosphorylation of mTOR (mechanistic target of rapamycin) which result in autophagy and a decrease in levels of collagen and ECM (Rangarajan et al., 2018[47]). Moreover, inhibition in AMPK activation by TGF-β results in mitochondrial dysfunction, which up-regulates the formation of mitochondrial ROS. Thus, a previous researcher has implicated TGF-β inhibition and activation of AMPK as an alternative therapeutic strategy to ameliorate organ fibrosis. Hesperidin is documented as a potent activator of AMPK (Rizza et al., 2011[48]) and in the present investigation also, the administration of hesperidin significantly ameliorated BLM-induced silencing of AMPK via inhibition of TGF-β levels which might have further exerted antioxidant, anti-inflammatory and antiapoptotic potential effects. The transmission electron microscope (TEM) analysis of lung tissue from hesperidin treated animals also supported this notion where BLM-induced mitochondria damage was augmented by hesperidin treatment.

Intratracheal instillation of BLM induces significant aberrations in histologic and cellular architecture of pulmonary tissue revealed by visualizing histological and transmission electron microscopic analysis of lung tissue from BLM control mice (Kandhare et al., 2015[23]). These alterations mimic the pathological features of clinical IPF which include excessive deposition of ECM protein such as collagen, shrunken mitochondrial cell nucleus, hyperplasia of type II pneumocytes (Kalayarasan et al., 2013[21], 2008[22]; Schuller-Levis et al., 2009[52]; Sriram et al., 2009[54]). In the present investigation administration of hesperidin inhibited BLM-induced aberrations of lung architecture, which was reflected by a decrease in perivascular and peribronchial collagen deposition in lung tissue determined using Masson trichrome stain and Picro-Sirius red stain.

Currently, corticosteroids as potent anti-inflammatory reagents have been widely used for the management of pulmonary fibrosis. However, their certain side effects and relief in the fraction of patient limit their implications in IPF treatment. Remedies of herbal origin are gaining more attention for the treatment of pulmonary fibrosis (Kilic et al., 2014[30]; Verma et al., 2013[56]). Moreover, the efficacy and safety of hesperidin has been well-established clinically in type 2 diabetes and overweight individuals (Eghtesadi et al., 2016[12]; Salden et al., 2016[50]). Thus, hesperidin may provide an alternative therapeutic regiment of natural origin for the management of IPF as well as an adjuvant to ameliorate the side effects induced due to BLM during the treatment of various carcinomas.

## Conclusion

**Figure 1:**
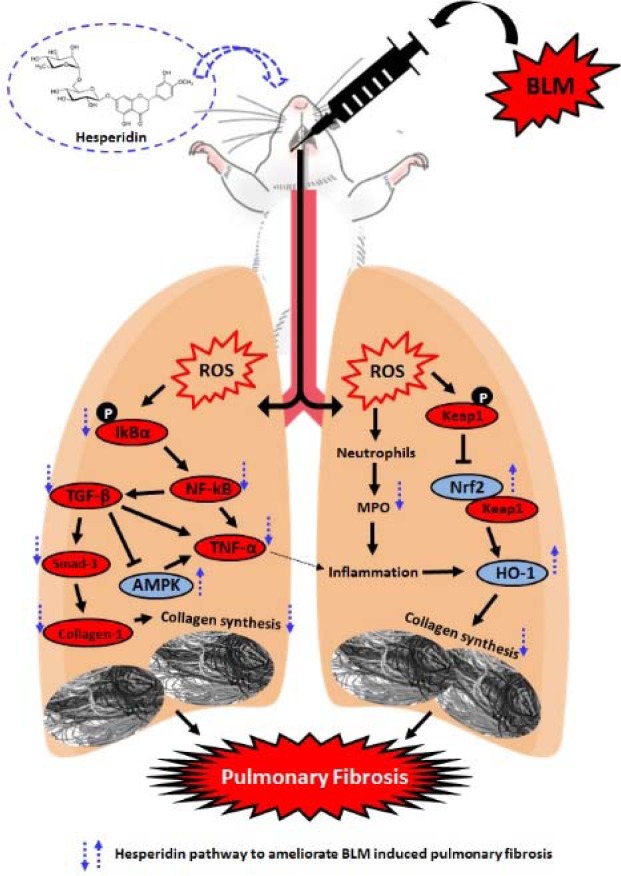
Graphical abstract

**Figure 2:**
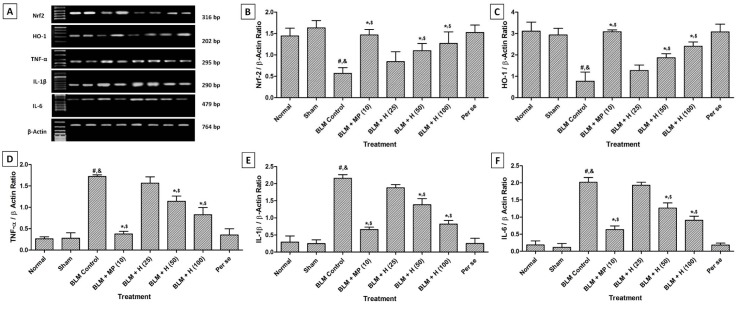
Effect of hesperidin on alterations induced by BLM in lung Nrf2, HO-1, TNF-α, IL-1β and IL-6 mRNA expression of rats (A) on day 14, quantitative representation of mRNA expression of Nrf2 (B), HO-1 (C), TNF-α (D), IL-1β (E) and IL-6 (F). Data were analyzed by One-Way ANOVA, followed by Tukey's multiple range post-tests. p < 0.05 when compared with: sham group (&), normal group (#), each other (MP or hesperidin) group ($), and BLM control group (*). BLM: Bleomycin, MP: Methylprednisolone, HO-1: Heme oxygenase 1, ILs: interleukin, Nrf2: nuclear factor E2-related factor 2, TNF-α: tumor necrosis factor-alpha. (n=4)

**Figure 3:**
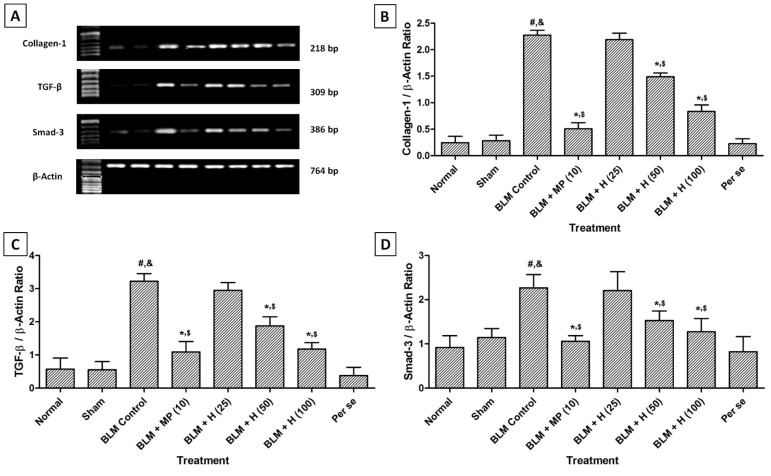
Effect of hesperidin on alterations induced by BLM in lung Collagen-1, TGF-β and Smad-3 mRNA expression of rats (A) on day 28, quantitative representation of mRNA expression of Collagen-1 (B), TGF-β (C) and Smad-3 (D). Data were analyzed by One-Way ANOVA, followed by Tukey's multiple range post-tests. *p < *0.05 when compared with: sham group (&), normal group (#), each other (MP or hesperidin) group ($), and BLM control group (*). BLM: Bleomycin, MP: Methylprednisolone, Smad-3: Mothers against decapentaplegic homolog-3, TGF-β: Transforming growth factor-β. (n=4)

**Figure 4:**
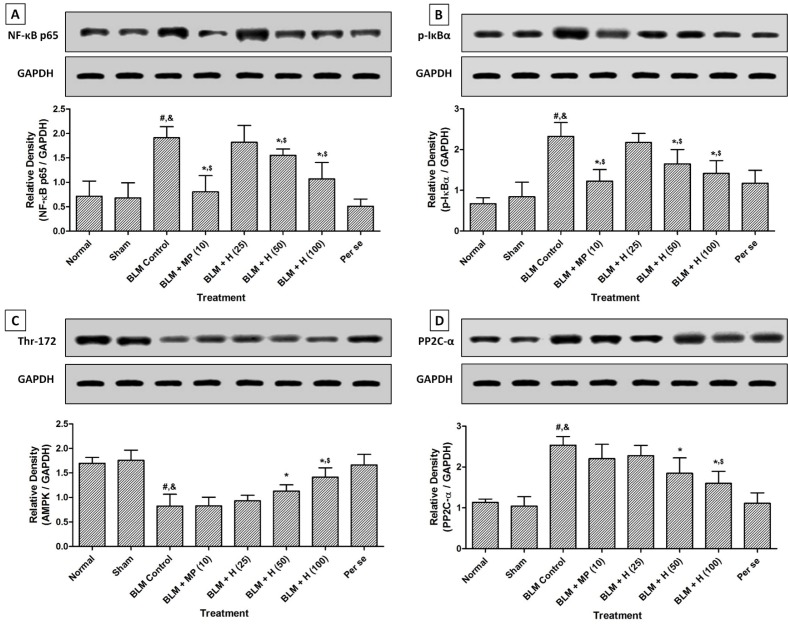
Effect of hesperidin on alterations induced by BLM in lung NF-κB (A), IκBα (B), AMPK (Thr-172) (C) and PP2C-α (D) protein expression of rats on day 28. Data were analyzed by One-Way ANOVA, followed by Tukey's multiple range posttests. p < 0.05 when compared with: sham group (&), normal group (#), each other (MP or hesperidin) group ($), and BLM control group (*). BLM: Bleomycin, NF-κB, nuclear factor-kappa B; IκBα: nuclear factor of kappa light polypeptide gene enhancer in B-cells inhibitor-alpha; Thr-172: Threonine-172 within the catalytic subunit (alpha) of AMPK; PP2C-α: Protein phosphatase 2C-Alpha; GAPDH: Glyceraldehyde 3-phosphate dehydrogenase. (n=4)

**Figure 5:**
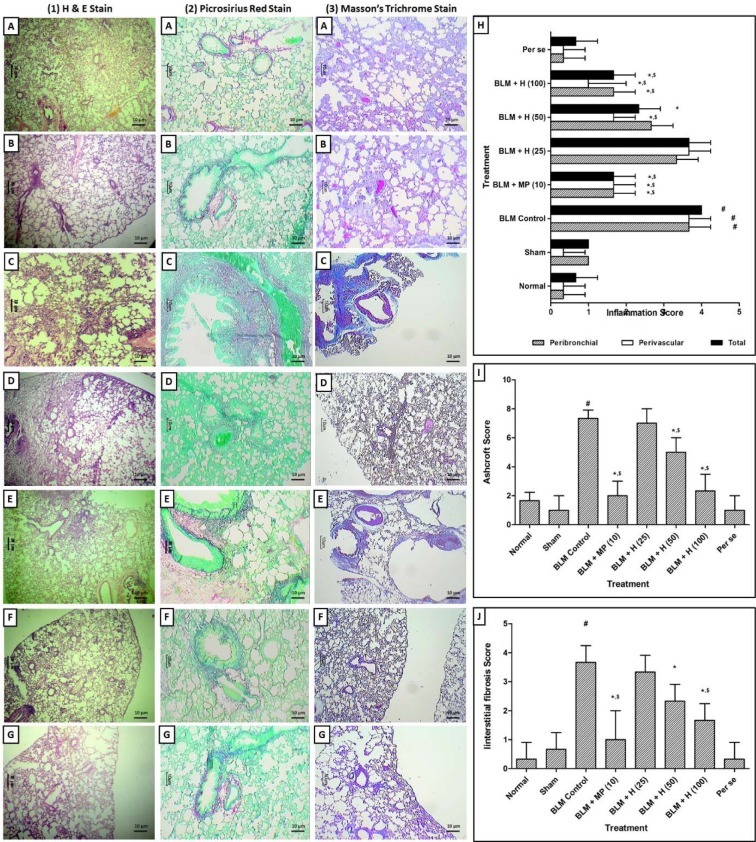
Effect of hesperidin on alterations induced by BLM in lung and airway histology of rats. Photomicrograph of sections of lungs of normal (A), sham control (B), BLM control (C), MP (10 mg/kg) treated (D), hesperidin (50 mg/kg) treated (E), hesperidin (100 mg/kg) treated (F) and Perse treated (G) rats. Lung H&E staining (1A-1G) on day 14, Lung Picro-Sirius red staining (2A-2G) on day 28 and Lung Masson's trichrome staining (3A-3G) on day 28. Effect of hesperidin on alterations induced by BLM in lung airway inflammation score (H), Ashcroft Score (I) and interstitial fibrosis score (J). Data were analyzed by One-Way ANOVA followed by Tukey's multiple range post-tests whereas data of the peribronchial, perivascular inflammatory scores, Ashcroft score, and interstitial fibrosis score were analyzed using non-parametric one-way ANOVA followed by Kruskal-Wallis test for post hoc analysis p < 0.05 when compared with: sham group (&), normal group (#), each other (MP or hesperidin) group ($), and BLM control group (*). (n=3)

**Figure 6:**
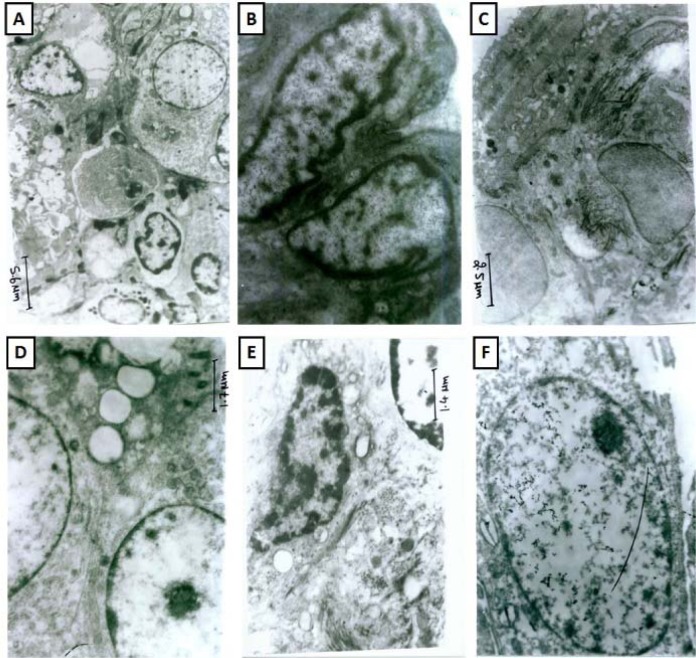
Effect of hesperidin on alterations induced by BLM in lung ultrastructural changes of rats. Photomicrographs of lung from representative animals, normal (at 3474 X) (A), sham control (at 11580 X) (B), BLM control (at 7720 X) (C), methylprednisolone (10 mg/kg) treated (at (11580 X) (D), hesperidin (100 mg/kg) treated (at 13510 X) (E), and per Se treated rats (at 11500 X) (F).

The results of the present investigation demonstrate that hesperidin alleviates bleomycin-induced pulmonary fibrosis in a biphasic manner. The antifibrotic potential of hesperidin is mediated through the inhibition of TGF-β1/Smad3/AMPK and IκBα/NF-κB pathways which ameliorate the modulation of oxido-inflammatory markers (Nrf2 and HO-1) and pro-inflammatory markers (TNF-α, IL-1β, and IL-6) to reduce collagen deposition during pulmonary fibrosis (Figure 1(Fig. 1)).

## Conflict of interest

The authors declare no conflict of interest.

## Figures and Tables

**Table 1:**
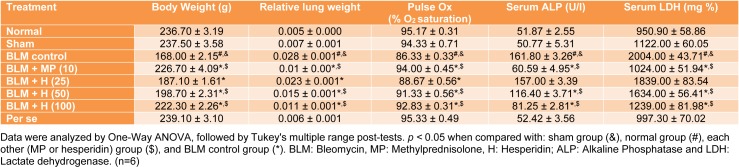
Effect of hesperidin on alterations induced by BLM in body weight, lung index, pulse Ox, Serum ALP and LDH on day 28

**Table 2:**
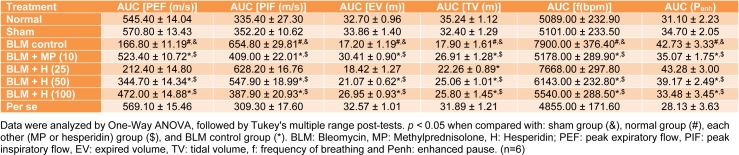
Effect of hesperidin on alterations induced by BLM in lung function test on day 28

**Table 3:**
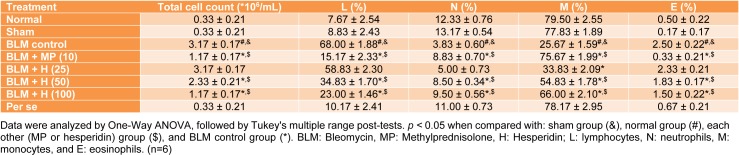
Effect of hesperidin on alterations induced by BLM in BALF differential cell count on day 14

**Table 4:**
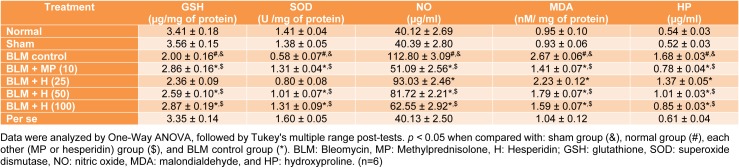
Effect of hesperidin on alterations induced by BLM in the level of BALF oxido-nitrosative stress and HP on day 14

**Table 5:**
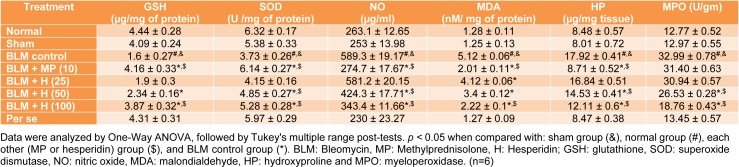
Table 5: Effect of hesperidin on alterations induced by BLM in the level of lung oxido-nitrosative stress, HP and MPO on day 14
